# Mini-open periacetabular cementoplasty for periacetabular bone metastasis: a report of two cases

**DOI:** 10.1007/s13691-024-00731-0

**Published:** 2024-11-02

**Authors:** Masahiro Kirisawa, Tomoaki Torigoe, Yasuo Yazawa, Takuya Watanabe, Yuho Kadono

**Affiliations:** 1 Department of Orthopaedic Oncology and Surgery, Saitama Medical University International Medical Center, 1397-1 Yamane, Hidaka, Saitama, 350-1298 Japan; 2 Department of Orthopaedic Surgery, Saitama Medical University, Moroyama-cho, Japan; 3 Department of Orthopaedic Surgery, Symphony Clinic, Utsunomiya, Japan

**Keywords:** Periacetabular bone metastases, Surgery, Mini-open periacetabular cementoplasty (MO-PAC)

## Abstract

Bone metastasis in the periacetabular region usually causes severe pain and functional disability. Some surgical procedures, such as the Harrington surgery and percutaneous cementoplasty, have been reported as treatment options for periacetabular bone metastases with limited efficacy. The former is highly invasive, while the latter may not allow the injection of a sufficient amount of cement. Here we report two surgical cases using a new modified surgical method (mini-open periacetabular cementoplasty: MO-PAC) consisting of tumor curettage and cementoplasty through a small incision.

## Introduction

While cancers metastasize to distant organs through both hematogenous and lymphatic routes, bone is one of the most common metastatic targets, observed in 5.13% of all cancer patients [1]. The pelvis is the third most common bone metastasis site, accounting for 10–20% of metastatic bone tumors [2]. Periacetabular metastasis often causes severe pain, gait disturbance, and eventual pathological fracture [3]. Although radiation therapy is a common treatment for those cases, it is sometimes difficult to achieve sufficient pain relief [4]. For these cases, hip replacement surgery options such as the Harrington procedure and percutaneous cementoplasty have been adopted to enable pain relief and preserve limb function [5–7]. Although surgical procedures such as total hip replacement can improve long-term pain relief and hip joint function, they are usually highly invasive and have a high complication rate. On the other hand, percutaneous cementoplasty can be carried out with minimal invasion. However, it is sometimes difficult to insert an adequate amount of bone cement when the osteolytic lesion is occupied by a solid and collagenous tumor tissue. In this study, we report a new modified surgical method (mini-open periacetabular cementoplasty; MO-PAC) that makes sufficient tumor curettage possible through a small incision and assures adequate bone cement packing in the aforementioned situation.

## Case reports

**Fig. 1:**
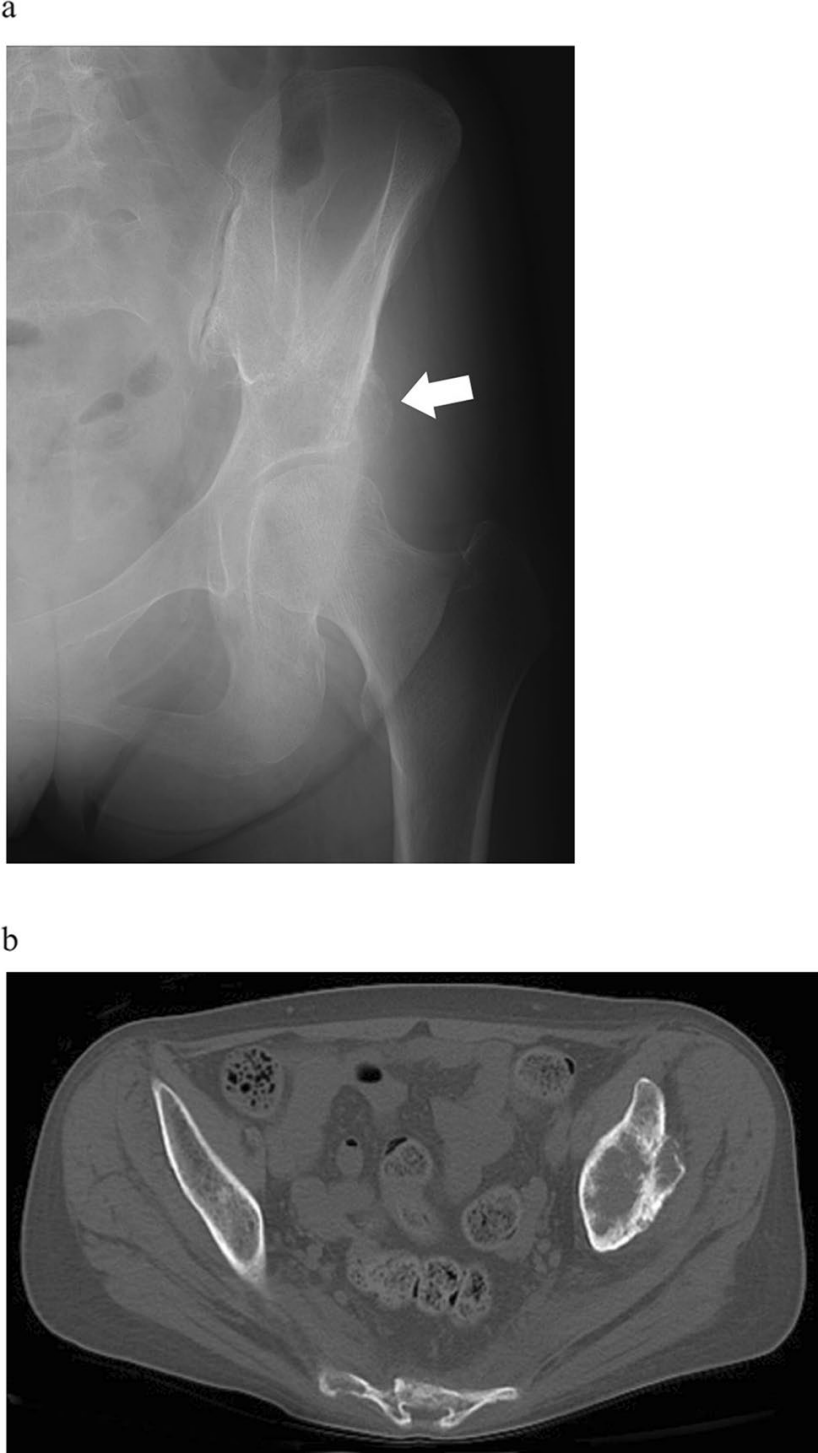
Anteroposterior X-ray of the pelvis shows osteolytic lesion in the left periacetabulum (arrow) (**a**). Plain axial CT shows osteolytic lesion in the left periacetabulum (**b**)

**Fig. 2:**
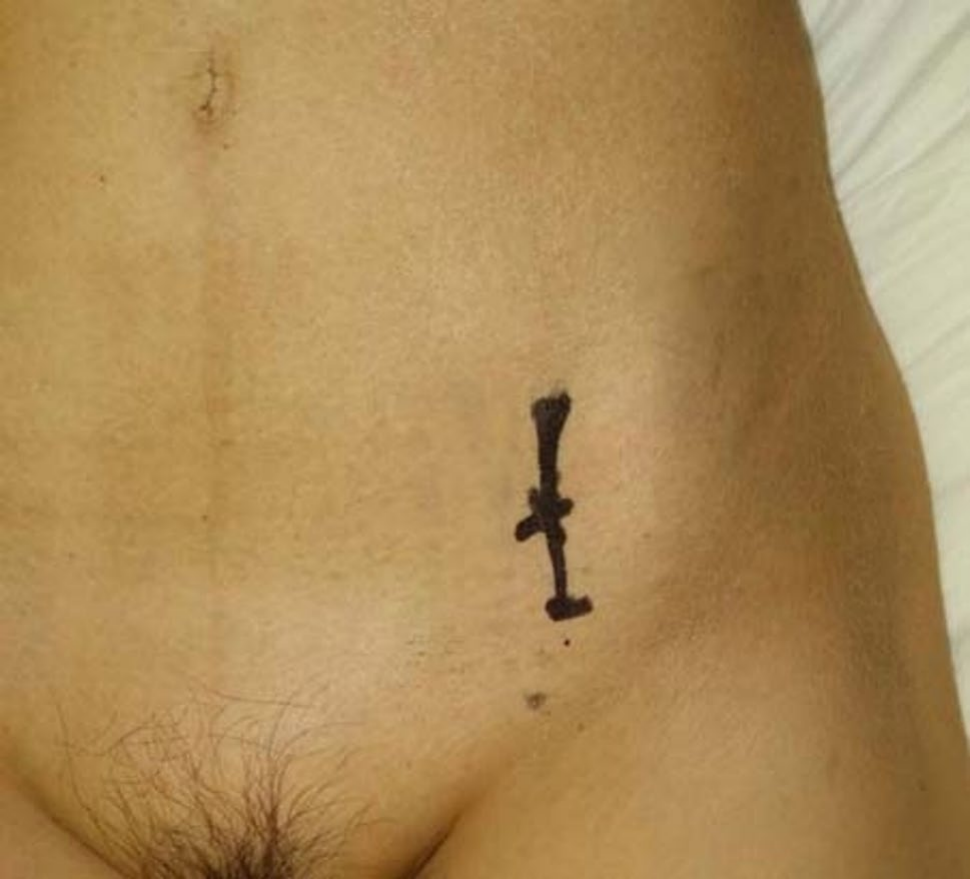
A small incision was made in front of the acetabulum

**Fig. 3:**
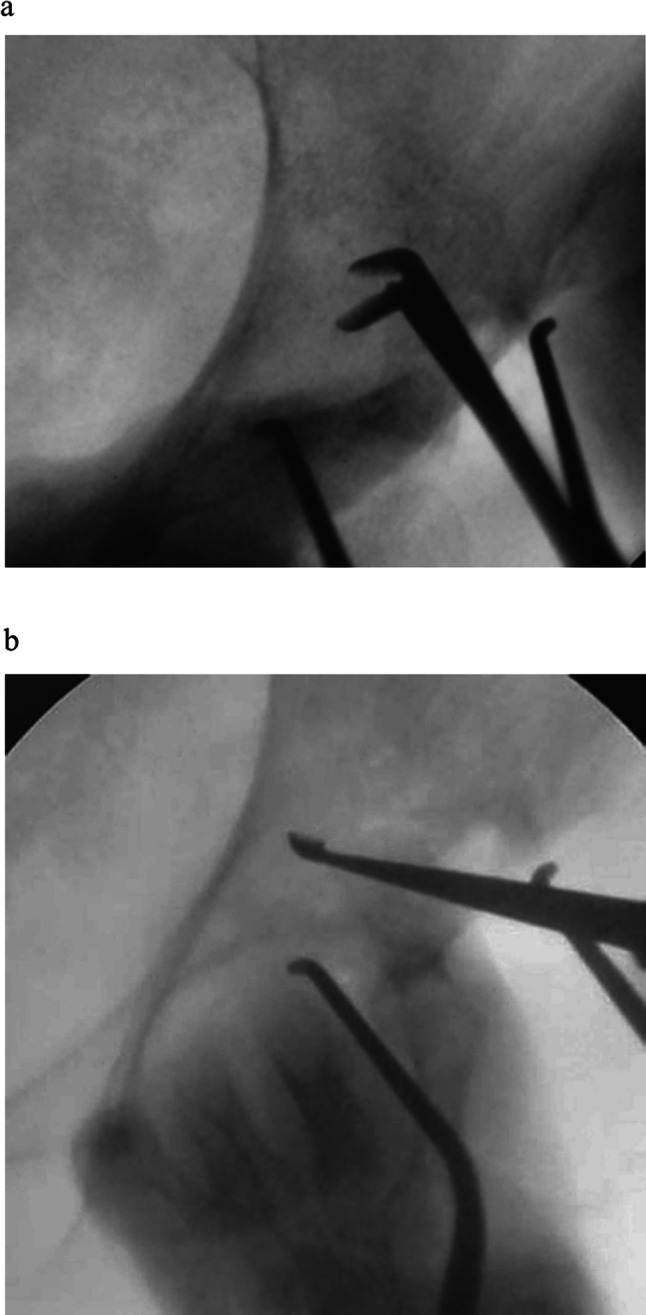
Curettage under image intensifier (**a**, **b**)

**Fig. 4:**
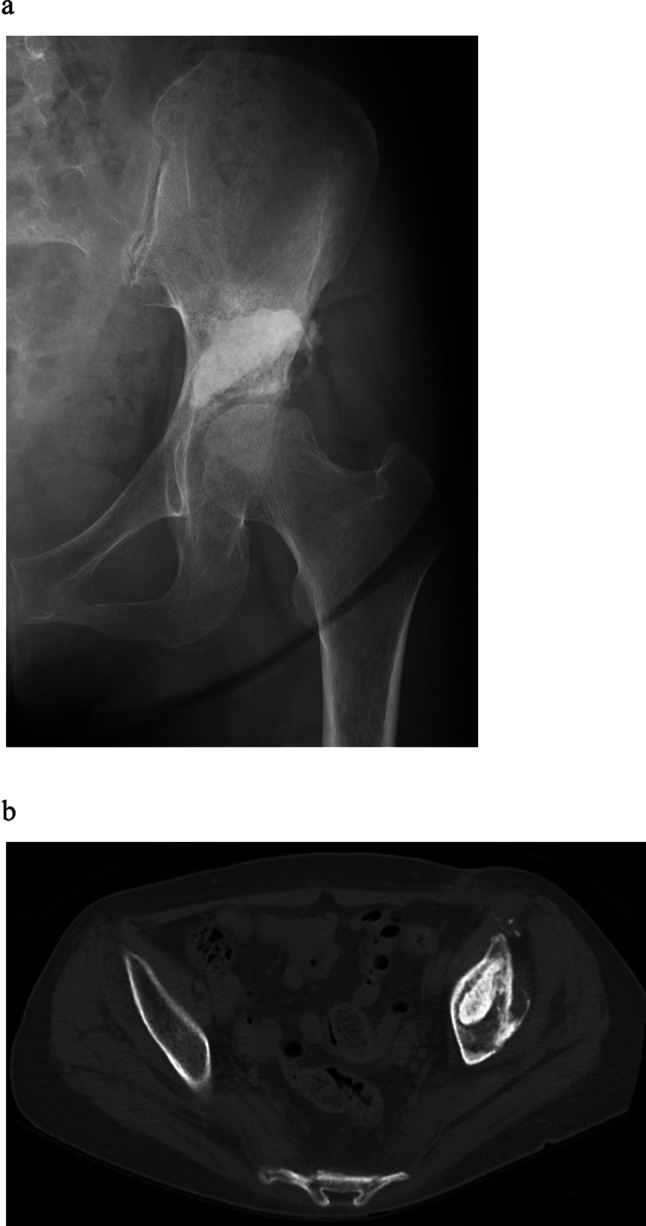
Postoperative X-ray of the pelvis exhibited that the lesion was filled with sufficient bone cement (**a**). Postoperative axial CT. Sufficient bone cement was filled (**b**)

Case 1. A 61-year-old woman was treated with chemotherapy for stage IV lung adenocarcinoma. However, the cancer progressed, and multiple bone metastases eventually emerged. The patient has also been administered denosumab. She had severe gait disturbance due to hip pain caused by left periacetabular bone metastasis (Fig. 1a, b). As the pain did not improve even after 25 Gy of irradiation, the attending physician estimated further chemotherapy was not applicable based on the patient’s poor performance status and was planning a palliative treatment. The modified Katagiri score at that moment was seven points and Harrington classification was evaluated as class I. However, through concomitant discussion with the patient to relieve the hip pain, MO-PAC was chosen and performed 3 months after the radiation therapy. During surgery, a small skin incision, approximately 4 cm, was made just anterior over the left acetabulum (Fig. 2). After making a small bone hole, approximately 1 cm in diameter, at the anterior inferior iliac spine, the tumor was curetted out under the monitoring of an image intensifier (Fig. 3a, b). Bone cement was then injected into the bone defect using a catheter tip syringe, and additional bone cement was inserted by applying manual pressure (Fig. 4a, b). The operation duration was 1 and 10 min, and the estimated blood loss was 15 ml. Immediately after surgery, the severe pain improved, and the patient was able to walk with full weight-bearing. There were no postoperative wound complications. At the evaluation 2 weeks after surgery, the Musculoskeletal Tumor Society (MSTS) score was 87% and the Toronto Extremity Salvage Score (TESS) improved from 67 to 87%. As the patient’s performance status had improved after surgery, the halted chemotherapy was resumed. During the following course, a contralateral femoral neck fracture occurred, and a hip hemiarthroplasty was performed. One year after MO-PAC surgery, there was no progression of periacetabular metastasis or femoral head deformity, and good function was maintained at 70% of MSTS score and 89% of TESS (Table 1).

**Table 1:** Clinical data of two patients

	Patient 1	Patient 2
Age/Sex	61/F	69/F
Primary cancer	Lung	Lung
Preoperative modified Katagiri score	7	7
Harrington classification	I	I
Surgical time (min)	70	80
Blood loss (g)	15	50
Preoperative TESS (%)	67	40
Postoperative TESS (%)	89	59
	(1-year after surgery)	(4-week after surgery)
Postoperative MSTS score (%)	70	77
	(1-year after surgery)	(4-week after surgery)
Pain	5	4
Function	3	1
Emotional acceptance	1	5
Supports	4	3
Walking	4	5
Gait	4	5

*F* female, *TESS* Toronto Extremity Salvage Score, *MSTS* Musculoskeletal Tumor Society

**Fig. 5:**
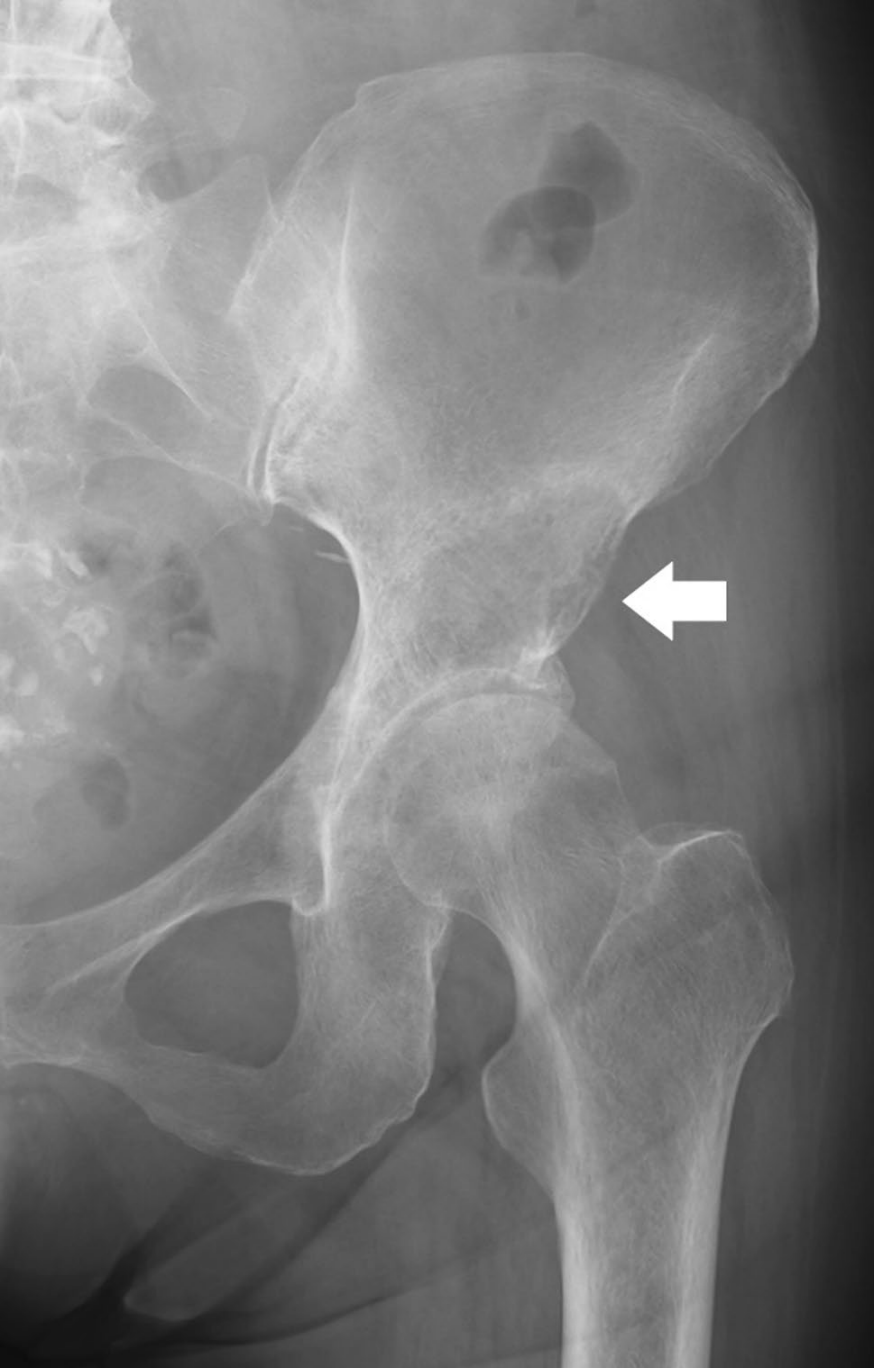
Anteroposterior X-ray of the pelvis shows osteolytic lesion in the left periacetabulum (arrow)

**Fig. 6:**
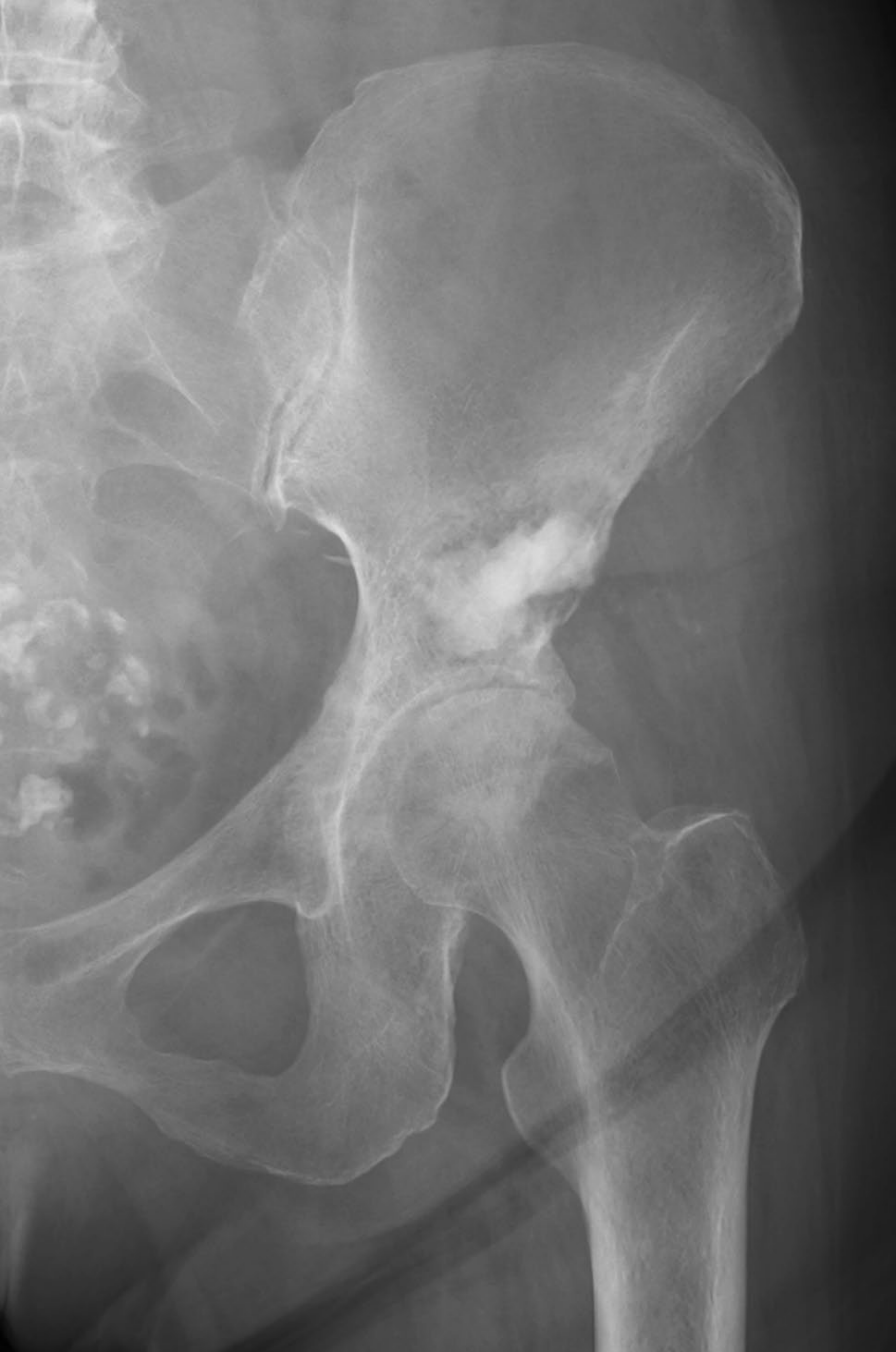
Postoperative anteroposterior X-ray of the pelvis shows the lesion was filled with favorable amount of bone cement

Case 2: A 69-year-old woman was treated with chemotherapy for stage IV lung adenocarcinoma. However, because a bone metastasis occurred in the left peri-acetabulum (Fig. 5), she experienced gait disturbance due to severe hip pain. The pain did not improve even after 25 Gy of irradiation and denosumab administration, and a palliative treatment was adopted afterward. When the patient was referred to our department due to worsening hip pain, modified Katagiri score was seven points and Harrington classification was class I. Similarly for this patient, MO-PAC was applied with the same surgical procedure as mentioned above at 4 months after radiation therapy. The operation time was 1 h and 20 min, and the estimated blood loss was 50 ml (Fig. 6). From the day after surgery, the pain improved, and the patient was ambulatory with full weight-bearing. There were no postoperative wound complications. At the functional evaluation 4 weeks after surgery, the MSTS score was 77%, and TESS improved from 40% preoperatively to 59% postoperatively. The patient was able to walk until she died 2.5 months after the surgery.

## Discussion

Periacetabular bone metastases commonly cause severe pain and adversely affect the activities of daily living [8]. In such instances, non-surgical treatments such as radiotherapy and bone modifying agent are generally applied as initial bone management. However, Callstrom et al. reported that pain relief was not obtained in 20–30% of cases after radiotherapy [9]. In our institution, 25 Gy of irradiation dose was commonly performed for bone metastases from lung cancer to relieve pain. However, there have been some cases in the literature in which the pain relief was insufficient. If the symptoms do not improve with these treatments, minimally invasive intervention or surgical treatment may be considered. However, the appropriate treatment for painful periacetabular metastasis after non-surgical treatment remains controversial and should be carefully planned based on its location, size, and the oncological condition of each patient [10]. In patients with a predicted long-term prognosis, for example in patients with breast cancer, thyroid cancer or cases with oligo-metastasis, tumor resection surgery using a hip prosthesis with a reinforcement ring, otherwise known as the Harrington procedure, are preferable [11]. Less-invasive treatments including percutaneous cementoplasty, radiofrequency ablation, cryoablation are generally indicated for cases with an estimated poor prognosis [12–15].

While hip prosthesis surgery can maintain long-term pain relief and preserve lower limb function, it is usually highly invasive with a high complication rate [16]. One systematic review reported that the postoperative mortality rate of periacetabular metastasis was 3.3% and the complication rate was 19.5% [17]. In another series, the mortality rate within 3 months after hip arthroplasty was 7% [18]. The use of such procedures needs comprehensive discussion especially for patients with advanced cancer, poor general condition, and poor prognosis.

The Harrington procedure is a method in which Steinmann pins are inserted retrograde from the acetabulum into the ilium, cemented together with the acetabular component, and followed by a total hip arthroplasty. As this procedure is technically complicated and not suitable for patients with advanced cancer, the modified Harrington method was developed, in which the direction of the pin was changed and/or screw/plate fixation was used and is recently more commonly reported. Although postoperative limb function and pain improvement are considered to be ideal, a wide range of complication rate has been reported at 6–53% based on systematic reviews [19–21]. Hence, the indication of this procedure also requires careful consideration [22].

In contrast, percutaneous cementoplasty for periacetabular bone metastasis, classified as a minimally invasive treatment, involves percutaneous injection of low-viscosity bone cement into osteolytic metastases [6, 7]. It has an ability to acquire immediate skeletal strength and provide pain relief. In addition, the skin incision is minimal and infection risk becomes very low. The reported mean Numerical Rating Scale (NRS) is significantly reduced from 6.1 points before surgery to 3.2 points at 1 week and 2.1 points at 1 month after the surgery. Postoperative gait function was improved in 28% of patients and 40% of the patients maintained this function. As for complications, extraosseous cement leakage was seen in 36% and pulmonary cement embolism in 11% [23]. There have been many reports on percutaneous cementoplasty, and most of them have similar results on good pain relief [24–26]. However, if a lesion has a high amount of collagen matrix, it is sometimes difficult to inject a sufficient volume of bone cement [27]. The cement volume that can be injected percutaneously is reported to be approximately 30% of the bone defect and the volume does not increase even after radiofrequency ablation [28]. It has been reported that cases with insufficient cement filling tend to experience less pain relief [29]. The durability of percutaneous cementoplasty is still controversial. The duration of pain relief has been reported to range from 7.3 months to more than 1 year [23, 30]. Even if pain relief is acquired initially, insufficient cement filling may be a cause of its low durability. Therefore, in cases with bone lesions occupied with solid and collagenous tumor matrix, percutaneous cementoplasty might not be suitable and open curettage and cementation surgery would be necessary. However, metastases from thyroid, kidney, or hematopoietic carcinoma tend to be less collagenous and more hemorrhagic, and consequently percutaneous cement injection may be preferable. [31].

While simple open curettage and cementation surgery has been a popular procedure for skeletal lesions and has also been demonstrated in many metastatic cases, this procedure for periacetabular lesions is still not routinely performed [32–34]. An article has reported 35 periacetabular metastases and 26 curettage and cementation surgeries that were performed with an average operating time of 168 min and a total of 3,150 ml of estimated blood loss [35]. This highlights the difficulty of the curettage surgery for this lesion.

There is a report of a cementoplasty and screw fixation performed through a 3-cm skin incision, and it was termed “minimally invasive” [36]. As our two cases required a 4-cm skin incision and 1 cm of cortical fenestration, we have termed it “mini-open” to indicate that it is more than minimally invasive but significantly less than the standard open method.

MO-PAC uses an anterior approach, through a small skin incision and a split of the iliopsoas muscle to directly reach the anterior site of the periacetabulum over a short distance. A small cortical fenestration of about 1 cm is then made at the anterior inferior iliac spine, allowing the tumor to be scraped off and sufficient cement to be injected. The major advantage of the anterior approach is that it provides easier access to the lesion compared to the lateral or posterior approaches. Disadvantage is that if the lesion is small and located at the posterior column, the anterior approach may compromise the mechanical strength of the periacetabulum. A lateral approach has a disadvantage of requiring a larger skin incision due to separation of the thick gluteal muscle and a greater distance to the periacetabulum. MO-PAC can be carried out with only an image intensifier guide and does not require intraoperative computed tomography (O-arm). Therefore, it can be performed in most standard medical facilities.

MO-PAC is a surgery that may be classified between percutaneous cementoplasty and simple open curettage and cementation surgery. Although there may be many cases that have undergone similar procedures in other medical facilities, to our knowledge, there have been no reports that clearly describe this procedure in detail. The significance of the naming of MO-PAC is to remind surgeons that there is another option of cementation surgery for the periacetabular metastasis. Cases of Harrington class I will be good candidates. Our two cases underwent this procedure without preoperative embolization, because hemorrhage during curettage was estimated to be minimal due to the solid nature of lung cancer tissue and preoperative irradiation fibrosis. However, in bone metastasis with abundant blood flow, such as renal cell carcinoma, hepatocellular carcinoma, and thyroid cancer, it is necessary to evaluate the blood flow of the periacetabular bone metastasis by contrast-enhanced CT before surgery, and preoperative embolization is indicated if necessary. We have been indicating the modified Harrington procedure for patients who are highly active, in good general condition, and with estimated long-term survival. MO-PAC is preferred for patients with short-term prognosis and/or poor general condition. However, as we only have the experience of two cases, the long-term durability of this procedure cannot be generalized. But still equal or longer durability is expected as that of percutaneous cementoplasty.

In conclusion, MO-PAC is within a spectrum of curettage and cementation surgery, and sub-classified as a type of minimally invasive surgery. We currently recommend this procedure for patients with painful periacetabular metastasis after radiotherapy, Harrington class I, low activity, and an estimated short prognosis.

## Data Availability

The data that support the findings of this study are available from the corresponding author upon reasonable request.
